# Knowledge Is (Still) Key: Awareness to Shape Trends in Telemedicine Use during the Pandemic Based on Management Perceptions and Implementation Systems

**DOI:** 10.1155/2023/4669985

**Published:** 2023-12-06

**Authors:** Nada I. Hawa, Tri E. B. Soesilo, Nuraeni Nuraeni

**Affiliations:** ^1^Research Cluster of Interaction, Community Engagement and Social Environment, Sekolah Ilmu Lingkungan, Universitas Indonesia, Jakarta Pusat 10430, Indonesia; ^2^Sekolah Ilmu Lingkungan, Universitas Indonesia, Jakarta Pusat 10430, Indonesia; ^3^Fakultas Kesehatan Masyarakat, Universitas Indonesia, Depok 16424, Indonesia

## Abstract

**Objectives:**

The digital revolution has brought rapid developments to the health sector. People were taking advantage of telemedicine technology during the COVID-19 pandemic. Telemedicine is highly recommended during a pandemic because it will reduce the transmission rate of viruses, and it is considered adequate and low-cost. However, a fundamental challenge still occurs; most people need to be used to telemedicine technology. Presumably, inadequate education and lack of experience regarding the use of telemedicine are obstacles for society in utilizing telemedicine.

**Methods:**

This study is aimed at determining the factors that influence the use of telemedicine. It focused on variables such as data confidentiality, administration, and knowledge to measure potential factors that pushed people to utilize telemedicine. We used a quantitative approach, using multivariate analysis, namely, simple linear regression. Most of our respondents are people aged 18-30 years young.

**Results:**

All respondents stated that administration factors in the implementation of telemedicine were good. Through the Chi-square test, the data safety factor has no effect (*p* value =0.090 or >0.05) on telemedicine implementation, while the knowledge factor has a significant effect on telemedicine implementation with a *p* value =0.043 (<0.005). The multivariate analysis explained that the knowledge variable influenced telemedicine use with a *p* value =0.033 (<0.05), meaning it contributed 1.624 times to telemedicine.

**Conclusion:**

This study discusses the factors that influence the use of telemedicine. The study's results explain that the knowledge variable is the most significant factor influencing telemedicine use. *Knowledge* is an intellectual property that everyone must have to capitalize on with telemedicine. A lack of knowledge will become an information gap and a barrier for someone to reach new tools/technologies.

## 1. Introduction

Advances in technology bring increasingly sophisticated aspects to the health sector. Telemedicine is an evidence-based practice resulting from technological developments [1]. Three pillars shape the telemedicine trend. The first is remote patient monitoring (RPM), which means remote patient control. The second is teleconsultation, namely, consultation between patients and health workers, for example, via web videos. The third is direct monitoring from home [2]. The advantage of telemedicine is that it is easy for patients to consult with doctors [3] and saves time and costs [4–7]. In addition, telemedicine can provide services via web video between service providers or health workers and patients in various remote areas [8]. Therefore, this approach can increase openness about the disease between service providers and patients because the younger generation finds telemedicine consultations more comfortable than consulting directly with health workers [9].

Telemedicine is increasing, especially during the COVID-19 pandemic, and there is great hope for the public to continue to consult with health workers [10]. Contactless consultations can ensure a safe relationship between patients and healthcare workers [11] and help avoid cross-infection [12]. Our research was conducted in Indonesia because Indonesia is known for the rapid spread of the COVID-19 virus among the people [10]. In a way, Indonesia, as a developing country, has specific social dynamics that determine the process of adopting a relatively new technological approach, which in this case refers to telemedicine. Therefore, the application of telemedicine during a pandemic needs to be studied by considering various factors in this field of study. A study by Hawrysz et al. explained that during the COVID-19 pandemic, patient satisfaction with telemedicine increased [13]. In a similar statement, providing online treatment is the best intervention during the COVID-19 pandemic, which can reduce the risk of transmission of COVID-19 [14]. However, testing these assumptions in the context of our research would be interesting. Here is a little more explanation regarding the condition of telemedicine in Indonesia. Currently, Indonesia has 17 telemedicine services, namely, Aido Health, Alodokter, Getwell, Good Doctor, Halodoc, Homecare24, KlikDokter, KlinikGo, Lekasehat, LinkSehat, Mdoc, Milvik Dokter, ProSehat, Sehatq, TrustMedis, Vascular Indonesia, and YesDok [15].

The current debate is centered on the issue of the lack of information and education that hinders people from accessing telemedicine technology. Telemedicine is used to increase digital literacy and technological resources [16]. Increasing digital literacy for the community can be done by providing education/information. Therefore, telemedicine can be applied to a broader field of health. Telemedicine allows health workers to treat children, refer patients to health services, treat patients virtually, or conduct consultations [17]. Based on previous research, the obstacles to using telemedicine are age and educational level [18–20]. The older the age, the longer it takes for individuals to receive new technology, and parents prefer to consult directly with health facilities rather than telemedicine [20].

At the same time, education level is a telemedicine obstacle because research also finds that the lower a person's education, the lower that person's knowledge, which is called a digital literacy deficit. Another weakness of telemedicine is that health workers cannot carry out physical examinations in person, so they are limited in providing emergency services. In addition, users need stable connectivity to access telemedicine services [7, 17]. However, there is still work to be done, particularly regarding differences in preferences based on knowledge and practical telemedicine applications. Our study contributes to exploring and predicting variables in sustainable telemedicine adoption.

## 2. Materials and Methods

### 2.1. Study Design and Sampling

This study was conducted in Surabaya City, East Java Province, Indonesia. This study uses a quantitative approach with a cross-sectional design. Surabaya, the capital city of East Java, is home to people of various ethnicities. The locals are joined by foreigners who came from other areas and settled in the area, which has resulted in the area being densely populated. Consequently, the spread of the COVID-19 virus is rapidly happening in this city.

**Figure 1:**
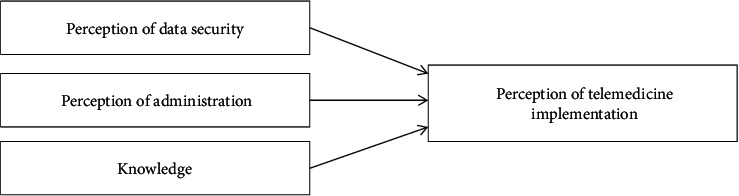
Research model.

Previous research has been conducted on telemedicine using a retrospective observational study method because of doubts arising during diagnosis and therapeutic management [21]. Meanwhile, this study is aimed at determining the factors that influence the use of telemedicine. It is essential to generate a perspective on which factors should be considered important and worthy of attention to implementing telemedicine technology in the healthcare sector, such as user knowledge [16, 22, 23] and perceptions regarding data security [24, 25] and administration in telemedicine practices [26, 27]. The conceptual framework for this research can be seen in Figure 1.

**Hypothesis 1 .**

Perception of data security influences the perceptions of telemedicine implementation.

**Hypothesis 2 .**

Perception of administration influences the perceptions of telemedicine implementation.

**Hypothesis 3 .**

The knowledge factor influences the perception of telemedicine implementation.

### 2.2. Sample Size Determination and Sampling Procedure

We used a simple random sampling method and selected respondents based on several inclusion criteria: telemedicine service users and smartphone users. We adjusted the inclusion criteria to the research objective, namely, the determining factors for the use of telemedicine, where people generally access telemedicine via smartphones. The number of respondents in this study was 400 respondents. The entire research process was carried out for one year in 2022 after COVID-19 hit.

### 2.3. Measures

Based on previous research on the use of telemedicine in the community, it is still necessary to adjust preferences based on age, knowledge, and practice of implementing telemedicine. Therefore, our study contributes to exploring and estimating the variables related to continued telemedicine adoption. In addition, we also explore knowledge, administration, and data confidentiality factors in the performance of telemedicine services. On the knowledge factor, the questionnaire questions included the types of services available on telemedicine, how to use these services, and the reasons for using telemedicine applications. The knowledge factor questions are six questions. Meanwhile, regarding health administration factors, we asked three questions related to perceptions of the importance of ease of administration, the importance of recording service access history, and the importance of minimizing document requirements for telemedicine services through yes or no statements. Finally, on the data confidentiality factor, three questionnaire questions cover the responsibility for data confidentiality and perceptions of personal data security when using telemedicine services.

Before conducting an in-depth analysis, researchers coded the answers to classify the data and simplify the data entry process. The data then goes through a data entry process. All data underwent a normality test with the Kolmogorov-Smirnov test (*p* > 0.05 = normal). Thus, we found that the dependent and independent variable scores were not normally distributed (*p* value =0.001). Therefore, we used medians for cut-offs for all variables. The median value for the variable knowledge is 6, administration and data confidentiality is 3, and for the use of telemedicine is 24. This study only focused on a few independent variables, which are crucial in discussing telemedicine usage.

### 2.4. Data Analysis

The data in the questionnaire is inputted into the Microsoft Excel Worksheet (.xlsx) and then inputted into the SPSS 26.0 application to assess the findings. We utilized univariate analysis to explain the characteristics of respondents (such as gender, age, occupation, education, type of telemedicine used by respondents, frequency of telemedicine, and activities often carried out while using telemedicine). It is necessary to describe certain patterns and tendencies among telemedicine users. Bivariate analysis was used to determine the relationship between the dependent and independent variables, and the analysis used was Chi-square analysis. It could give a proper explanation regarding factors that pushed people to utilize telemedicine. Furthermore, to see the most dominant factors influencing the use of telemedicine, the researchers also used regression analysis.

## 3. Results

### 3.1. Demographic Features of Respondents

Based on the data we collected through questionnaires, most of our respondents were women, namely, 251 respondents or around 62.8%, while the rest were men, 149 respondents (37.3%). While on the age factor, generally, 18-30, namely, 321 respondents (80.3%), and respondents aged <18 years, 31-40 years, and >40 years, each less than 10%. Students dominate the respondents. We define a student as someone who goes through a learning process at all levels of formal education. A total of 340 respondents (85%) were students, 20 respondents (5%) were private employees, five respondents (1.3%) were self-employed, two housewives (0.5%), and two government officials (0.5%). Thirty-one respondents (7.8%) are not included in the previously mentioned occupational groups. Respondents in general (365 respondents or 91.3%) had a high school education, 8.3% (33 respondents) had a bachelor's degree, and the remaining 0.5% (2 respondents) had a master's degree (Table 1).

One of the platforms in telemedicine services still being used by the public is Halodoc (39.8%), a franchise provided by the private sector. Meanwhile, the services provided by the government, namely, mobile JKN, still need to be used by the public in this study, namely, only 0.3% of the total respondents. Respondents also use Alodokter (5.3%), KlikDokter (1%), and other franchises that require explicit identification (2.5%). The majority of respondents stated that they no longer used telemedicine (51.3%), while 10% of respondents stated that they still used it once a month, and the frequency was more than once a month and less than or equal to three times a year, each less than 10%. They all have experience using telemedicine, primarily for consulting doctors (30%), while their other activities are reading health articles (26.5%), buying medicine (9.5%), laboratory testing (2%), getting COVID-19 medicine for free (1.6%), and other telemedicine activities (30.5%) (Table 1).

### 3.2. Factors Influencing Perceptions of Telemedicine Implementation

We also performed the 2 × 2 table relationship analysis in Table 2. We conducted a Chi-square test between the variables of perception of administration, data security, and respondents' knowledge of the variable of perception of implementing telemedicine. Regarding data security actors, 199 respondents (49.75%) stated that their data security was secured. Among them, 47.7% of them stated that the implementation of telemedicine was good, and 52.3% said it was poor. Whereas 50.25% of respondents stated that the security of their data was not guaranteed, 56.7% of them stated that the implementation of telemedicine was good, while the rest stated the opposite. The relationship between the two variables has a *p* value of 0.09 (*p* value >0.05). Thus, the two variables do not have a significant relationship.

Researchers could not conduct a Chi-square statistical test on administrative factors because all respondents (100%) stated that administration was good. However, the percentage of respondents' opinions regarding the implementation of telemedicine is slightly different. Namely, 52.3% said it was good, and 47.8% said it was poor. Meanwhile, the knowledge variable related to the application of telemedicine has a *p* value of 0.043 (*p* value <0.05) or is significantly related. Dominant respondents had good knowledge (290 respondents), where 49% considered the implementation of telemedicine to be good, and 51% stated that the implementation of telemedicine was poor. Meanwhile, 110 respondents had poor knowledge, of which 60.69% of respondents considered the implementation of telemedicine to be good, and 39.1% of respondents stated that the implementation of telemedicine was poor. The next step is to select significant variables (*p* value <0.05) based on the Chi-square test results and enter them into the regression test. Thus, the regression test can only be carried out on one variable that meets the significant requirements, namely, the knowledge variable.

The regression analysis results in Table 3 show that the knowledge variable produces a *p* value of 0.03 (*p* value <0.05). In other words, knowledge has a significant relationship with perceptions regarding the application of telemedicine. The odds ratio value indicates that people with better knowledge will have the opportunity to utilize telemedicine services by 1.624 times (95% confidence interval). At the same time, the value of *B* indicates that knowledge has a positive correlation with the use of telemedicine.

## 4. Discussion

Telemedicine is one of the targets of world technological progress [28, 29] in aspects of alternative health services [30, 31] that are safe, efficient, and effective in meeting patient needs [24]. Telemedicine is suitable for conditions that are not urgent and do not require an extensive physical examination [32]. Healthcare workers can use technology to contact patients, such as the telephone [33], or other telecommunications media, such as electronic health records, social alarms, and online client portals used in medicine [34].

The application of telemedicine involves a complex integration between information technology and healthcare services that enables service providers to diagnose, consult, and treat patients without geographic boundaries. By leveraging online platforms and dedicated applications, telemedicine revolutionizes traditional healthcare delivery while maintaining high medical standards. In the context of a global pandemic and increasing demand for medical services, telemedicine is increasingly becoming an essential solution in overcoming barriers to access and improving the quality of medical services in general [6, 14, 35, 36].

Specifically in Indonesia, this study's results indicate that telemedicine use still needs to be increased. Currently, there is only one telemedicine franchise that dominates the Indonesian market, namely, Halodoc. This finding implies that telemedicine, the style of today's digital devices, is not yet as prevalent among the public. Services of telemedicine include administration, information, and treatment. Our research results also show that most respondents were women, and most came from the younger generation, especially those aged 18-30. This finding is similar to other studies, with the average age of research respondents being 29 years [32] and other findings that telemedicine is suitable for the younger generation [17, 20], who are also more familiar with the use of technology and are open-minded in accessing modern health services. This study's results also show the younger generation's interest in telemedicine applications.

This condition is different in people with old age. Our findings align with Jaron in Poland, who found that the age group 60 years and over showed reluctance to access teleconsultation [37]. Telemedicine can only partially replace inpatient services, especially in people with old age. Telemedicine services should adapt according to patient needs to reduce obstacles and problems in this system, such as making long-distance visits to reassure the public [38]. Older patients can also use simple technological devices such as tablets which can make it easier to connect with doctors accompanied by counseling patients and preparing health experts or unique technology to deal with elderly patients and help them how to use digital devices [37].

Health services that use more than one service modality, such as distance medicine, have the potential to report higher utilization rates of health services compared to those that only have one service modality [33]. However, our study found that its application still faces challenges, particularly in the context of our research in Indonesia. Our research has found that this is because several barriers, including knowledge, can stop people from using this technology. While today's technology, namely, telemedicine, can be associated with the younger generation, this was not always the case, as the barriers identified can create problems in the future. In this study, we found that knowledge is one of them. The results of our research explain that the better a person's knowledge, the better access to telemedicine, with our respondents being high school graduates (91.3%) with student status (85%) or, in other words, continuing their education. This finding is in line with other research, which found a need for more telemedicine-related knowledge among research respondents who were also students [39, 40].

One study stated that telehealth investments could increase the utilization of youth health services and help maintain relationships with adolescents during shocks, including during a pandemic or other conditions that can limit physical access to health services [33]. The success of any use of new technology in the health sector depends on the user's knowledge, perception, and skills regarding the concept of program implementation [41]—digital skills of health workers and skills of patients [33]; attitude; and environment [40]; physical limitations of the patient; patient's cognitive limitations; sociodemographic and clinical characteristics of patients [34]; limitations of service provider diagnosis and management; perception of high costs (especially for mental health services) [32]; and language barriers [42]. There are specific legal, cultural, and social challenges [43]. Medical personnel are also concerned about possible emergencies due to limited patient visualization during telephone consultations. However, with telemedicine, health workers can perform triage, refer patients to health services, treat patients virtually, or perform a consultation [17]. Approaches to implementing telemedicine services vary, mainly based on the patient's attitude, age, and adaptation needs [38]. In such circumstances, telemedicine can potentially exacerbate health inequities [37]. Policymakers must identify barriers among special populations to using telemedicine. They should initiate interventions to overcome these barriers while adapting them to the needs of this group [37]. Telemedicine can increase the number of patients who do not complete necessary laboratory and other tests [29].

Previous research found that there is avoidance of using relatively new services because people do not trust and benefit from these services [44–46]. On the other hand, other studies have found that telemedicine can answer health problems before and even after the COVID-19 pandemic [47–49]. Therefore, the government should provide education to the public about the use of telemedicine. Our findings show that the government-provided telemedicine platform is less popular with the public. Thus, as the primary stakeholder, the government must prepare new efforts. Other studies have also noted that people still perceive telemedicine as an “alternative” way to meet their health needs [2, 3, 46, 48, 49].

According to another study, the number of respondents with good knowledge as much as 67.4% of respondents had also received health information from the Internet as much as 23.6%, and 60.59% of respondents had visited telemedicine platforms. Respondents stated that they often exchange information about the COVID-19 virus via telemedicine as much as 75.3% and usually use communication services via telephone as much as 30.6% [50]. This finding is also consistent with other research, which found that the higher the respondent's education, the higher the person has knowledge. At the same time, our research results found that the *p* value was 0.015, which means that education affects the level of knowledge. Adequate knowledge is essential to encourage someone to try something new. Therefore, people must find ways to increase digital literacy to keep up with the increasingly sophisticated times [36, 51].

Due to our findings, we suggest increasing capacity building for citizens, positive medical or academic training, and continuing medical education for more complex diagnoses, which also play a small but integral role in video and web conferencing. This training can become a global learning forum by recording and broadcasting the learning sessions so that other doctors or students can access them for free. Additionally, facilitating the transfer of medical knowledge from sources in research centers using audiovisual and digital tools reduces the information gap for healthcare providers in underserved hospitals [47]. In other words, increasing digital literacy among young adults will create a tendency to take advantage of new technologies, especially those that benefit them.

Based on the results of our study, the data security variable has a *p* value of 0.090 (>0.05), which means it does not affect the use of telemedicine. However, data security is an essential variable in telemedicine technology. In order for users to feel comfortable and continue to use the telemedicine platform, service providers must provide data security that is confidential and accurate [3, 18, 49, 52]. One form of data security that a telemedicine platform must facilitate is access control. Access control is a critical security solution to restrict unauthorized users from accessing one's services. Access control is an essential tool for healthcare related to patients' health and living conditions. In addition to access control, service providers must also utilize cryptographic technology. Cryptography has been widely used as an essential security solution to ensure several security requirements, such as confidentiality and data integrity [53].

Indeed, telemedicine has many advantages but can only partially replace the primary doctor-patient relationship. Some treatments would be more optimal in person, not just virtually. However, telemedicine is aimed at providing online counseling [54] and general healthcare services for various nonemergency general health conditions, including allergies, respiratory infections, skin rashes, sinus problems, migraines, and others [53–57]. Thus, it is necessary to expand the range of families or patients who can benefit and optimize patient care [58]. Ultimately, we still have to pay attention to the advantages and disadvantages of telemedicine. In short, telemedicine is more valuable for ensuring citizens' health than focusing on various diseases. During the pandemic, telemedicine provided a way for people to meet their medical needs, which implies that technology can solve some critical problems, especially during emergencies. Meanwhile, public awareness of health is critical. The government and multisectors must be able to carry out collaborative interventions to strengthen awareness [59]. Collaborating with the community is one strategy that can help increase awareness of participation [60]. Apart from that, it is also essential to focus on increasing knowledge. Increasing knowledge is essential because it is closely related to individual attitudes [61, 62], encouraging increased awareness. Without adequate knowledge, people tend not to use new technology, even though the technology can provide benefits.

## 5. Conclusions

The use of telemedicine has increased significantly during the COVID-19 pandemic. Our findings highlight knowledge as the most crucial variable. Good knowledge does not necessarily have implications for good perceptions of telemedicine implementation. In other words, telemedicine technology needs to be updated to attract its users' interest. Meanwhile, we also found telemedicine users with poor knowledge. Thus, education regarding this technology must be increased so that users are accustomed to using it. Knowledge of telemedicine will enable them to use it wisely. In addition, our research implies that health awareness fundamentally extends beyond knowledge of technology. With a proper understanding of the importance of health, society will look for ways to direct it toward it. In addition, social barriers in society cause their reluctance or inability to access valuable technologies such as telemedicine. Further research on telemedicine may explore these barriers. In addition, it is also essential for future researchers to focus on the effectiveness of telemedicine as a technology that functions to increase public health awareness and not only focus on the ability or effectiveness of telemedicine as a technology to cure disease.

## Figures and Tables

**Table 1:** Characteristics of respondents (*n* = 400).

Variables	Frequency	Percent
Sex		
Female	251	62.8
Male	149	37.3
Age (year)		
<18	24	6
18-30	321	80.3
31-40	21	5.3
>40	34	8.5
Occupation		
Student	340	85
Government officials	2	0.5
Private employees	20	5
Entrepreneur	5	1.3
Housewife	2	0.5
Others	31	7.8
Education		
High school	365	91.3
Bachelor's degree	33	8.3
Master's degree	2	0.5
What kind of telemedicine that they use?		
Halodoc	159	39.8
Alodokter	21	5.3
KlikDokter	4	1
JKN Mobile	1	0.3
Others	10	2.5
No longer use	205	51.3
The frequency of using telemedicine		
No longer use	211	52.8
Once a week	15	3.8
Thrice a week	9	2.3
Once a month	43	10.8
Thrice a month	30	7.5
Once a year	35	8.8
Thrice a year	7	1.8
Others	50	12.5
What do they do with telemedicine?		
Doctor consultation	120	30
Buy medicine	38	9.5
Reserve for laboratory test	8	2
Reading health articles	106	26.5
Get free COVID-19 medicine	6	1.6
Others	122	30.5

**Table 2:** The relationship between independent and dependent variables.

Variable	Perception of telemedicine implementation				Total		*p* value	Explanation
	Good		Poor					
	*N*	%	*N*	%	*N*	%		
Perception of data security								
Secured	95	47.7	104	52.3	199	100	0.090	Hypothesis 1 rejected
Unsecured	114	56.7	87	43.3	201	100		
Perception of administration								
Good	209	52.3	191	47.8	400	100	—	Hypothesis 2 rejected
Poor	0	0	0	0	0	0		
Knowledge								
Good	142	49.0	148	51.0	290	100	0.043	Hypothesis 3 accepted
Poor	67	60.9	43	39.1	110	100		

Cut-off with median.

**Table 3:** The important factor that determines telemedicine utilization.

No	Variable	*B*	*p* value	OR	95% CI
1	Knowledge	0.485	0.033	1.624	1.039-2.539
	Constant	-0.443			

## Data Availability

The data and material of this article are not publicly available due to ethical matters but are available from the corresponding author on reasonable requests.
